# Stressors experienced by informal caregivers: implications for home care for dependent older adults

**DOI:** 10.1590/0034-7167-2025-0052

**Published:** 2025-11-03

**Authors:** Joice Lourenço da Silva, Elen Ferraz Teston, Luciana de Alcantara Nogueira, Edileuza de Fátima Rosina Nardi, Maria Eduarda Pascoaloto da Silva, Leticia de Castilho Peralta, Maria de Fátima Mantovani, Sonia Silva Marcon

**Affiliations:** I Universidade Estadual de Maringá. Maringá, Paraná, Brazil; II Universidade Federal de Mato Grosso do Sul. Campo Grande, Mato Grosso do Sul, Brazil; III Universidade Federal do Paraná. Curitiba, Paraná, Brazil

**Keywords:** Home Health Nursing, Frail Elderly, Stress, Psychological, Caregiver Burden, Life Change Events., Cuidador, Cuidado Domiciliario, Anciano, Salud Familiar, Teoría de Enfermería.

## Abstract

**Objectives:**

to identify the stressors experienced by informal caregivers based on Betty Neuman’s Systems Model.

**Methods:**

this descriptive, qualitative study was conducted with informal caregivers of older adults registered and assisted by an institution in Presidente Prudente, São Paulo. Data were collected in October 2023 through audio-recorded semi-structured interviews and subjected to thematic content analysis.

**Results:**

the 12 participating caregivers were between 30 and 59 years old. Conditions such as sleep deprivation, physical exhaustion, lack of support, financial hardship, and compromised health are examples of stressors experienced, which impacted caregivers’ ability to cope with care demands without compromising their physical and emotional health.

**Final Considerations:**

interventions that promote balance, mental health, and strengthening caregivers’ support networks can help them find and use more effective coping and self-regulation mechanisms.

## RESUMEN

**Objetivos:**

identificar los factores estresantes que experimentan los cuidadores informales según el Modelo de Sistemas de Betty Neuman.

**Métodos:**

este estudio descriptivo y cualitativo se realizó con cuidadores informales de ancianos registrados y atendidos por una institución en Presidente Prudente, São Paulo. Los datos se recopilaron en octubre de 2023 mediante entrevistas semiestructuradas grabadas en audio y se sometieron a un análisis de contenido temático.

**Resultados:**

los 12 cuidadores participantes tenían entre 30 y 59 años. Condiciones como la privación del sueño, el agotamiento físico, la falta de apoyo, las dificultades económicas y la salud comprometida son ejemplos de factores estresantes que afectaron la capacidad del cuidador para afrontar las exigencias del cuidado sin comprometer su salud física y emocional.

**Consideraciones Finales:**

las intervenciones que promueven el equilibrio, la salud mental y el fortalecimiento de las redes de apoyo de los cuidadores pueden ayudarlos a encontrar y utilizar mecanismos de afrontamiento y autorregulación más efectivos.

## INTRODUCTION

In Brazil, between 2010 and 2022, the older population increased by 57.4%, reaching a total of 32.1 million individuals aged 60 or over, accounting for 15.8% of the total population^(1)^. Globally, the population aged 65 or over exceeded 10% in 2023 and could reach 16% by 2050^(2)^. These data pose economic and social challenges, since increased life expectancy, especially when accompanied by risky habits and behaviors related to health conditions, can lead to senility and, consequently, dependence on daily care^(3)^. Furthermore, the occurrence of external causes, especially in the adult population, has also been responsible for physical or cognitive disabilities and the consequent dependence on care, which increases the demand for constant support and specialized care^(4)^.

Sometimes, caregiving activities are offered within the family environment. However, financial constraints, in addition to moral and emotional commitments, often lead families to opt for informal caregivers, often family members without psychological preparation or formal training^(5)^. Furthermore, cultural issues, lack of access to support resources, and the absence of effective public policies to support caregivers increase the challenges these individuals face in their daily lives^(6)^.

Caring for a family member requires routine adjustments and coping with challenges that, without adequate support, can harm caregivers’ physical and mental health. These detriments manifest themselves in the form of stress, overload, and neglect of self-care^(7)^, contributing to the onset or worsening of illnesses. Furthermore, caregivers’ health conditions can compromise the quality of care provided^(7)^.

Brazilian public policies for informal caregivers remain limited and fragmented. While the Continuous Care Benefit program offers indirect support, isolated initiatives, such as the “Caring for Those Who Care” program in Florianópolis, stand out for providing psychological support and technical training^(8)^. The Brazilian Health System (In Portuguese, *Sistema Único de Saúde* - SUS) also contributes through primary care teams and Psychosocial Care Centers (In Portuguese, *Centros de Atenção Psicossocial* - CAPS), although the lack of national standardization for training initiatives highlights significant gaps. In this context, the proposal to create the Brazilian National Care Policy, still under consideration, reinforces the need for more consistent progress in this area^(9)^.

In this context, Betty Neuman’s Systems Model emerges as a holistic framework that understands the human being as an open system, constantly interacting with the environment and protected by lines of defense that foster resilience against internal and external stressors. Based on the levels of primary, secondary, and tertiary prevention, the model proposes not only health promotion but also minimizing the impacts of stressors and restoring balance^(10)^ through the implementation of structured interventions integrated with support networks, public policies, and self-care practices. When used appropriately, it allows for the comprehensive identification and meeting of individuals’ needs, including those of informal caregivers.

In Brazil, informal caregivers face significant challenges, amplified by factors such as emotional overload associated with a lack of preparation and adequate support, family conflicts, and a lack of social recognition, which constitute interpersonal stressors. In turn, the lack of comprehensive public policies and insufficient financial resources represent extrapersonal stressors, which exacerbate the negative impacts on these individuals’ health^(11)^.

In this scenario, the systemic approach offers a relevant perspective by considering that protective barriers can be compromised by the lack of support strategies or preventive actions, causing imbalances that result in adverse physical and mental health conditions^(10)^. Understanding the stressors faced by these caregivers allows us to understand how they impact their physical, emotional, and social defenses^(12)^. Furthermore, this knowledge can support the formulation and implementation of intervention strategies that involve social, emotional, and educational support, favoring the restoration and balance of caregivers, thus preventing the development of diseases and promoting their health and well-being^(13)^.

## OBJECTIVES

To identify the stressors experienced by informal caregivers in light of Betty Neuman’s Systems Model.

## METHODS

### Ethical aspects

The study was conducted in accordance with the ethical principles of Resolution 466/2012 and was approved by the Research Ethics Committee of the signatory institution. All participants agreed to participate in the study by signing the Informed Consent Form. To ensure their anonymity, in the presentation of results, excerpts of their statements are identified with the letter “E” (interviewee), followed by a number indicating the order of inclusion in the study.

### Theoretical-methodological framework

Betty Neuman’s Systems Model integrates concepts of open systems, psychology, and physiology, and understands the human being as a holistic system, in constant interaction with the environment and vulnerable to stressors that affect its balance^(10)^. In Brazil, its application in nursing research gained prominence in the 1990s, especially in the field of public health, becoming a relevant tool for analyzing stress factors and planning care interventions^(14)^.

The model classifies stressors into intrapersonal, interpersonal, and extrapersonal, facilitating the identification of specific needs and the implementation of interventions that strengthen individuals’ coping capacity^(10)^. In professional practice, its application occurs through a systematic assessment of patients’ condition and the development of personalized plans aimed at reducing stressors and promoting well-being^(14)^.

### Study design

This descriptive, qualitative study preceded the implementation of a nursing intervention based on Betty Neuman’s Systems Model for informal caregivers of dependent older adults. The study report was written using the criteria established in the COnsolidated criteria for REporting Qualitative Research^(15)^ as a supporting tool.

### Study setting

The study was conducted in the homes of older adults dependent on care, assisted by a public institution located in a municipality in the state of São Paulo. This institution offers outpatient and home healthcare services, as well as activities aimed at promoting socialization, autonomy, self-care, and self-esteem through a health promotion gym. The Universal Home Care Service for Older Adults, known as S.A.U.D.I., is intended for individuals who are bedridden or have reduced mobility.

Approximately 800 healthcare appointments are provided monthly. The institution relies on a multidisciplinary team consisting of two social workers, four nurses, five physical therapists, three nutritionists, a speech therapist, two psychologists, three geriatric physicians, a pharmacist, two physical education professionals, and three nursing assistants.

### Methodological procedures

Study participants were 12 informal caregivers of dependent older adults, selected by convenience during home visits conducted by the healthcare team between August and September 2023. The invitation to participate in the study was only extended after the lead researcher had participated in at least two visits, i.e., when a minimum relationship had already been established between her and the caregiver.

The previously defined inclusion criteria were being at least 18 years of age, having worked as an informal caregiver for the dependent older adult for at least three months, and demonstrating self-reported ability to understand and respond to the data collection instrument. No exclusion criteria were established.

### Data collection and organization

Data were collected in October 2023 through audio-recorded interviews, after obtaining consent, and scheduled in advance, based on participant preference and researcher availability. The interviews were conducted without the presence of members of the multidisciplinary healthcare team or the patients being cared for. They lasted 60 to 120 minutes, with each participant being interviewed only once.

During the interviews, a two-part interview guide was used: the first addressed caregivers’ sociodemographic and health characteristics; the second addressed aspects related to the caregiver role, such as time dedicated to the activity, older adults’ level of dependence, the difficulties faced in daily life, and the strategies used to overcome them.

All interviews were conducted by the same researcher, a nurse, master in family health and doctoral student in nursing, with previous experience in conducting qualitative research and who maintained only professional contact with participants.

### Data analysis

The interviews were transcribed in full, preferably on the same day they were conducted, and then subjected to thematic content analysis, following the three proposed stages^(16)^. In the pre-analysis, the data were organized and subjected to a cursory reading to initially identify relevant aspects. In the subsequent stage, material exploration, a detailed reading was performed, followed by coding the data into seven codes, which were grouped into three core meanings. Finally, in the stage of processing the results obtained and interpretation, after code classification and aggregation, one category and three subcategories emerged, which were interpreted and discussed in light of Betty Neuman’s Systems Model^(10)^, with inferences being made in relation to the findings.

The transcribed interviews were not validated by participants, due to the overload of domestic activities and care demands.

## RESULTS

Twelve caregivers aged between 30 and 59 participated in the study. Eight were daughters and four were daughters-in-law of older adults dependent on care. Five were married, four were single, two were divorced, and one was a widow. Two participants were away from work due to health issues, while the others had to leave their jobs to care for their dependent family members, with no prospect of reintegrating into the labor market because they lacked a support network or alternative means of assistance. The family income of seven participants ranged from one to two minimum wages; four lived on three to four minimum wages; and only one lived on five to six. It is noteworthy that none of the families received social benefits.

All caregivers used at least one medication regularly. Six used one to two medications daily; five used three to four; and one used five to six. When asked about their own health status, seven classified it as “fair”, three as “poor”, one as “good”, and one as “very poor”, with seven considering themselves stressed at the time of the interview. Reported health problems included anxiety (ten), hypertension (five), depression (five), back problems (four), type 2 diabetes (four), heart failure (one), and hypothyroidism (one).

### The hidden side of care: the physical and mental challenge of caring for others, for oneself, and for surviving stressors

This thematic category was structured based on data analysis, grouping the main stressors identified by participants. Data coding yielded three core meanings, which were organized into three subcategories: The conflict of caring for others as an interpersonal stressor; Self-care as a challenge: intrapersonal stressors; and Financial vulnerability as an extrapersonal stressor. These subcategories reflect the complexity of the informal care phenomenon and were interpreted in light of Betty Neuman’s Systems Model.

### The conflict of caring for others as an interpersonal stressor

This subcategory expresses the tensions that arise from relationships with dependent older adults, other family members, and care demands.

Sleep deprivation, physical exhaustion resulting from the constant demands of routine caregiving, the worsening of preexisting health conditions, and the emergence of new health problems were stressors reported by participants.

[...] *the end of the day comes, I’m just dust, with no energy for anything else. I can’t always handle everything I have to do, you know. The burden of caring for her is huge, and we feel exhausted* [...] *in the end, we don’t even sleep properly.* (E06)

*After she got sick, I never knew what it was like to sleep eight hours a night* [...] *the most I sleep in a night is about three hours.* (E02)

*Some days she sleeps well, but other days she doesn’t* [...] *since I sleep in the same room as her, I wake up very quickly to see what she needs. When I sleep, I’m afraid she’ll call me and I won’t wake up, you know, so I usually go to bed already worried.* (E10)

*I think her current health condition has also greatly affected her psychological state* [...] *which consequently has affected mine* [...] *because there are times when I think I won’t be able to handle it.* (E01)

*There’s no time left to do the things I want. There’s no time left* [...] *it’s total dedication, just for her, alone* [...] *not even for my husband lately.* (E02)

*The day goes by too quickly. Before I know it, I’m still in my nightclothes, and it’s already three in the afternoon, and I haven’t done anything but take care of her since I got out of bed.* (E03)

*Sometimes I think she could be more grateful for me changing my entire life and family routine to take care of her, but that doesn’t happen*. (E06)

[...] *I talked to him yesterday... dad, I’m tired physically, emotionally, spiritually, everything.* (E03)

### Self-care as a challenge: intrapersonal stressors

Intrapersonal stressors refer to the internal reactions triggered by the role of caregiver, including feelings of frustration, fear of illness, and impacts on self-esteem.

*She has some attitudes that really stress me out, and I need to hold myself back* [...] *but there are times when it makes me very anxious.* (E01)

Taking on the role of full-time caregiver destabilized some participants’ systems, making self-care difficult. Sometimes, the intense routine of caring for others makes it difficult for caregivers to set aside time for themselves and feel like they have not accomplished anything, even though they have accomplished a lot throughout the day.

*I’m afraid of having a mental problem, you know? Sometimes we see people dependent on sleeping pills* [...] *I was afraid of being dependent on these medications forever.* (E10)

*I confess that sometimes I’m afraid of going crazy, it’s so overwhelming, it’s exhausting to care for someone* [...] *and I feel very alone. In fact, I don’t even have anyone to talk to.* (E12)

*I’m not going to tell you that I don’t go out* [...] *but I worry about her all the time, so I think we don’t go out, that’s the general family thing. The family doesn’t go out peacefully anymore, we go out to dinner, lunch, there’s no* [...] *we can’t enjoy that moment as much, because we’re worried about the person who’s here.* (E02)

*Having time for myself, like going to the salon, is almost impossible* [...] *there’s no way I can leave the house and leave her here alone, and I don’t trust leaving a stranger to take care of her, like* [...] *she doesn’t talk, I’m afraid of mistreatment, you know* [...]. (E09)

*When I have any time left, I want to rest because I’m very tired. I also have my health problems* [...] *I need to rest, because otherwise I can’t take it anymore, I’ll end up getting sick too.* (E02)

High levels of stress and frustration resulting from the caregiving routine impact caregivers’ self-esteem, which ultimately compromises their perception of self-worth and psychological well-being.

*I think being a caregiver for someone we know, a family member, is more stressful because we know them.* (E12)

*Today, I feel more stressed. My self-esteem is very low because before, I could go to the gym, I could take better care of myself, and now I can’t anymore.* (E02)

*There are days when I get frustrated thinking that I have to do so much for my mother and that she won’t be the same again. No matter what I do, she won’t walk again, for example. That’s frustrating* [...] *all the work and no results.* (E10)

Similarly, the difficulties and adaptations faced in relation to learning new skills, as well as situations that promote individual growth throughout the process of caring for a dependent person in the home environment, were highlighted.

*I didn’t know how she would progress with this disease, to be honest. I was surprised, because I thought the condition progressed very quickly* [...] *I don’t know if that’s true, I’d never cared for someone with Alzheimer’s; everything I know I learned from her on a daily basis.* (E12)

*I knew nothing about the disease. I watched videos online and on social media, and I learned. Little by little, I realized that each person’s progress varies, and I learned to adapt to our family’s needs.* (E09)

*Initially, social media was the biggest help to me in learning about this disease. I researched online how the disease progressed because I didn’t have direct information from professionals at first.* (E08)

### Financial vulnerability as an extrapersonal stressor

Participants highlighted that economic difficulties and problems related to the support network constitute extrapersonal stressors that exacerbate the challenges faced in everyday life.

*Our expenses here are very high, right? It’s not easy. We worry a lot about the bills* [...] *because some of her medications we can’t get through the SUS. Everything goes through the pharmacy, right? So, it’s very expensive.* (E02)

*Currently, I don’t have any income, because before, I could go out for private trips. Now, I rarely manage to do so, like, let’s say I earn 40 reais a month* [...] *but let’s say nothing, because that’s nothing.* (E03)

*She only earns minimum wage, so it’s not enough to buy everything she needs, unfortunately. There are months when I have to choose what I’ll stop paying to try to keep up with her basic needs.* (E05)

However, even in the face of challenges, strategies used by caregivers to mitigate the effects of stress in their daily routine were highlighted.

*When I’m really sad, I play gospel music on my phone to try to feel better, you know* [...] *when I listen to praise songs, it seems to renew my energy*. (E10)

*But when I feel stressed, I pray, I praise; I have my little phone, I put it there, I play praise songs* [...] *I say my prayers.* (E02)

[...] *when she does something that stresses me out, I try to avoid caring for her for a while, I leave the room to breathe* [...] *but sometimes, I end up taking it out on those around me.* (E06)

## DISCUSSION

The stressors identified in this research, interpreted in light of Betty Neuman’s Systems Model, comprised the thematic category “The hidden side of care: the physical and mental challenge of caring for others, for oneself, and for surviving stressors”. The findings reveal the complexity of the challenges faced by informal caregivers, highlighting the predominance of women in this role, especially daughters and daughters-in-law, the renunciation of professional life due to the lack of support, and the physical and emotional impacts resulting from the intense caregiving routine.

Issues such as sleep deprivation, overload, solitary search for information, lack of leisure time, and financial constraints further exacerbate these women’s vulnerability. These factors highlight the urgent need for structured social and institutional support strategies aimed at strengthening support networks and promoting caregivers’ well-being. While interventions of this nature would not change the underlying, structured conditions that sustain this situation, they can minimize the stressors caregivers experience daily and help them identify potential actions that promote well-being and quality of life.

Organizing the findings into a single thematic category with three subcategories reflects the interdependence between the types of stressors identified, reinforcing Neuman’s systemic approach. The integration of the core meanings allowed us to view caregiver burden as a multidimensional phenomenon, involving interpersonal, intrapersonal, and extrapersonal aspects, which feed into each other and compromise the caregiving system’s defenses.

Thus, in this structurally challenging context, characterized by the predominance of female informal caregivers, specific age groups, kinship ties and substantially limiting socioeconomic conditions, the implementation of strategies aimed at mitigating the effects of this reality becomes important^(17)^. In this context, Betty Neuman’s Systems Model emphasizes the context of comprehensive care, especially when seeking to understand the dynamic interaction between humans and the environment.

The interpersonal stressors identified in this study corroborate the results of a study conducted in South Korea, which highlighted stress, physical exhaustion, and intense time commitment to caregiving as key factors in the burden of informal caregivers^(18)^. These findings reinforce the urgency of specific strategies focusing on secondary prevention for the early identification of these risk factors for these individuals’ physical and mental health, who face challenges such as fatigue caused by daily tasks, such as mobilizing patients and administering medications, in addition to the lack of time for self-care, aggravating emotional exhaustion^(11)^.

Considering the fundamentals of Betty Neuman’s Systems Model^(10)^, the constant need to balance family and professional responsibilities constitutes an intrapersonal stressor. In turn, extrapersonal stress stems from a lack of social and financial support, which weakens caregivers’ defenses, compromising their health and role performance. Interpersonal stressors, in turn, arise from direct contact with the dependent family member and the lack of recognition for caregivers’ efforts, generating feelings of frustration and emotional isolation. These factors reduce the effectiveness of protective barriers, especially in contexts of limited public health support, which intensifies the effects of emotional and physical overload. Difficulty accessing medication and necessary care further aggravates this process, increasing caregivers’ vulnerability to illness and exhaustion.

According to Neuman, the breakdown of the normal line of defense in the face of recurring stressors requires actions at all levels of prevention^(10)^. In this study, we observed that caregivers frequently resort to coping strategies centered on faith, silence, or personal sacrifice, which highlights the lack of structured interventions at the primary level. The lack of formal support also compromises secondary and tertiary prevention actions, making the caregiving system more susceptible to progressive exhaustion.

In this study, psychological stressors associated with patients’ health conditions and the constant care demands generate emotional overload, loneliness, and fear of mental breakdown. Chronic stress weakens resilience and hinders the ability to cope with the challenges of caregiving, contributing to feelings of anxiety, fear, and helplessness in the face of accumulated responsibilities^(19)^.

In turn, physical overload and lack of time for self-care, coupled with interpersonal challenges such as responsibility for caring for others and a lack of support, can also significantly impact caregivers’ mental health. The interaction between external and internal stressors creates a vicious cycle, in which the lack of adequate support intensifies stress, culminating in caregivers’ physical and psychological illness, and consequently, makes it difficult to recognize the need for help.

The limited or even complete absence of support for informal caregivers is not a problem unique to Brazil. A phenomenological study conducted on Ilha da Madeira, Portugal, also revealed that these caregivers face a shortage of home care and financial support, as well as a lack of psychological counseling and training programs. The authors highlighted that the lack of an adequate support network can compromise caregivers’ physical and emotional health and intensify the stress levels they experience^(20)^.

In this regard, we suggest expanding existing initiatives in Brazil, such as Primary Care teams and CAPS, to provide emotional support through support groups and psychological therapy. The expansion of home care and palliative care services within the SUS, combined with the implementation of respite programs and the strengthening of community support networks, can contribute to promoting caregivers’ comprehensive health. Furthermore, it is essential to broaden the debate on promoting these individuals’ mental health, considering the adoption of strategic actions that include guidance on psychological issues and stress management. Such initiatives must include both caregivers and their families, promoting open dialogue about the difficulties experienced and alternatives to strengthen lines of defense, resilience, and overall well-being^(19)^.

The importance of strengthening support for informal caregivers was highlighted in an intervention, using an educational game as a support tool, conducted with 19 caregivers in Portugal. The results demonstrated effectiveness in improving emotional and educational support, leading the authors to conclude that the use of this strategy contributed to strengthening caregivers’ defenses, resulting in more balanced and efficient performance in caregiving activities^(21)^.

Participants in this study reported devaluing their own needs due to a lack of time for themselves and a feeling of insignificance, despite being directly responsible for the numerous, exhausting care demands. This scenario reflects a cycle of overload and self-effacement, resulting from interpersonal and extrapersonal stressors that destabilize physical, emotional, and social health^(19)^. The constant prioritization of others over oneself intensifies feelings of emptiness and frustration, revealing a progressive erosion of protective barriers, especially regarding mental health and personal-life balance.

Care demands interfere with enjoyable activities, such as spending time with one’s spouse and enjoying leisure time. Replacing self-care and social interactions with exhausting tasks directly affects emotional health, weakening interpersonal and family bonds. This imbalance between self-care and care for others compromises caregivers’ protective mechanisms, increasing their vulnerability to stress and physical and emotional exhaustion, making it difficult to maintain well-being and the balance necessary to face the challenges of caregiving^(19)^.

Given this scenario, promoting self-care can reduce some of the impact of care demands, although its implementation depends on factors such as financial status and access to resources. In more favorable contexts, it is possible to hire formal caregivers and seek emotional support. In vulnerable situations, family and community networks can offer free support. Rest periods, redistribution of tasks, and guidance on time management are viable strategies to strengthen caregivers’ well-being, regardless of socioeconomic status.

Implementing these measures would not only strengthen caregivers’ ability to protect themselves from stressors, but would also contribute to reducing external factors that harm their psychological and physical well-being^(11)^. These aspects are important, as the caregivers in this study highlighted the complexity and emotional strain involved in caring for a family member, which, despite being carried out with affection, brings profound tensions due to the closeness and emotional bond.

In this context, communication also plays a crucial role, as caregivers often have to act on instinct or deduction due to a lack of adequate guidance. Transmitting information without considering caregivers’ level of understanding intensifies feelings of helplessness and frustration, worsening psychological stress. Furthermore, the perception that their efforts are not resulting in significant improvements in their loved one’s care contributes to emotional exhaustion, undermining motivation and the ability to cope with adversity^(22)^.

Many caregivers face difficulties adjusting to new tasks, especially when they lack knowledge about the disease in question. Learning occurs through everyday experience and information acquired online and on social media. While essential, this process reveals a lack of specialized guidance, overwhelming caregivers with information to be assimilated independently and not always scientifically based. Therefore, more targeted professional support is important to enhance the skills and personal development necessary for caring for others, which, in turn, strengthens the ability to cope with suffering^(23)^.

Hence, promoting self-care helps caregivers face daily challenges, but its viability depends on factors such as income and access to resources. Families with better resources can hire formal caregivers and seek emotional support. Those in vulnerable situations may or may not rely on family and community networks. Therefore, strategies such as rest, task sharing, and time management are essential to preserving caregivers’ health.

Participants reported using strategies to cope with daily stress, such as listening to specific music or even singing, praying, and taking short breaks. These practices, especially spiritual ones, offer emotional support, providing a respite from the stresses of caregiving and allowing a reconnection with the meaning of life and spirituality^(24)^. Prayer and praise were highlighted by participants as resources that help reduce stress, relieve tension and promote emotional tranquility, functioning as coping resources that strengthen caregivers’ resilience, especially when caring for family members with chronic illnesses.

Religious practices tend to be highly valued by older adults, especially in healthcare settings. It is common these days for older adults to care for dependents, although this was not the case for the participants in this study. For older adults in community groups, quality of life involves more than just nutrition and physical activity-it includes good family relationships, spirituality, and religiosity, which, according to them, offer comfort, strength, and hope, helping them cope with limitations, loss, and stress^(25)^.

Temporarily removing oneself from a stressful situation can also be seen as an adaptive strategy to preserve mental health^(22)^. While escape does not solve the underlying problem, it offers caregivers much-needed time to rebalance their emotions and avoid impulsive reactions that could harm their relationship with the person receiving care. These coping strategies, which seek to restore caregivers’ emotional and mental energy, are crucial, especially given the burden imposed by the intense caregiving routine. However, they are not sufficient to mitigate long-term stressors, highlighting the importance of more structured interventions that offer ongoing psychological and social support, in addition to appropriate medical care^(26)^.

These experiences highlight the importance of continuing education for caregivers, as well as access to clear information about chronic diseases and coping strategies^(27)^. It can be inferred that the implementation of training and support programs, developed jointly by the healthcare network-including Primary Healthcare, hospital services, and Home Care Services-can improve the quality of life of caregivers and those they care for. While the use of tools such as the internet is valuable, it is essential that this support be complemented by practical and specialized guidance.

Using analytical models to identify the stressors faced by informal caregivers offers an opportunity to transform a challenging context into a more balanced and supportive reality. This approach enables the creation of interventions that ensure continuity and quality of care, while protecting caregivers’ health and well-being^(19)^. With ongoing and appropriate support, it is possible to mitigate the effects of chronic stress and improve the ability to cope with the physical, emotional, and social demands of this role.

These measures, by strengthening coping mechanisms, help restore or preserve the caregiving system’s normal defenses, promoting physical and emotional balance. According to Neuman’s model^(10)^, this ongoing support can work both at the primary level, by preventing stress from worsening, and at the tertiary level, by aiding the system’s recovery after a crisis.

Thus, consolidating the findings into a single thematic category does not represent a limitation, but rather a methodological choice based on the interconnection between stressors and the systemic nature of informal care. By simultaneously addressing physical, emotional, relational, and social aspects, the analysis reveals the inseparability of the factors that impact caregivers’ health, reinforcing the usefulness of Neuman’s model as a framework for analysis and intervention.

### Study limitations

A possible limitation of this study lies in the fact that the sample consisted of only 12 caregivers, all located from the older adult registry in a single institution, which may have limited the variability in the identification of stressors and the impossibility of validating transcripts by participants due to care overload.

### Contributions to nursing

The study expands understanding of the risk factors for stress among informal caregivers of dependent individuals in the home environment, elucidating the internal and external dimensions of the stressors experienced by this population group. By focusing on the emotional, physical, and social impact of caregiving, the study raises awareness of a vulnerable group often overlooked in the care of dependent individuals.

Using Betty Neuman’s Systems Model to analyze caregivers’ experiences offers a robust theoretical framework that can be applied in clinical practice to identify stressors and their consequences for caregivers. By considering the different levels of defense and resistance mechanisms, this model allows nursing to develop targeted and personalized intervention strategies for each phase of caregivers’ experience.

By identifying the main stressors and their impact on caregivers, the study contributes to the development of health promotion programs targeted at this population, focusing on stress prevention and improving quality of life. Self-care strategies and the implementation of emotional support interventions, for instance, can be integrated into community and home nursing work, having a direct impact on caregivers’ health.

## FINAL CONSIDERATIONS

The study, based on Betty Neuman’s Systems Model, reaffirmed the complexity of the stressors experienced by informal caregivers and the coping mechanisms adopted by caregivers as self-regulation strategies, including prayer and music. Such practices provide emotional relief, although they prove insufficient to significantly reduce high levels of stress, particularly with regard to physical and emotional exhaustion.

Overloaded tasks, social isolation, and a lack of financial and emotional support, combined with physical and mental health difficulties, intensify caregivers’ routines. The lack of recognition and support, combined with worsening conditions such as anxiety and depression, compromises their ability to cope, generating feelings of frustration and overwhelm. These factors highlight the need for interventions that promote balance, mental health, and strengthen caregivers’ support networks.

The development of public policies and support programs that offer emotional support, guidance on stress management, and self-care strategies is necessary. Creating a more robust support network, including psychological, social, and financial assistance, is essential to reduce the negative impact of informal care and preserve the health and well-being of caregivers, strengthening their ability to cope with care demands without compromising their physical and emotional health.

Future studies should focus on implementing and assessing personalized interventions for informal caregivers, considering diverse sociocultural contexts and individual characteristics. Research on emotional resilience, remote support technologies, and therapeutic groups is promising. It is also important to assess public policies that offer financial and emotional support, as well as the effectiveness of integrative practices in promoting caregivers’ mental health and well-being. While such interventions cannot modify the underlying, structured conditions that sustain this scenario, they can minimize the stressors experienced daily by caregivers and help them identify potential actions that promote their well-being and quality of life.

## Data Availability

The research data are available only upon request.
