# Supplementary material for: Stressors experienced by informal caregivers: implications for home care for dependent older adults
Source: Rev Bras Enferm. 2025 Nov 3;78(Suppl 2):e20250052. doi: 10.1590/0034-7167-2025-0052 (PMC12584952; doi:10.1590/0034-7167-2025-0052)
Supplement: Translation

# Translation: Estressores vivenciados pelo cuidador informal: implicações para o cuidado domiciliar ao idoso dependente

**DOI:** 10.1590/0034-7167-2025-0052pt

**Authors:** Joice Lourenço da Silva, Elen Ferraz Teston, Luciana de Alcantara Nogueira, Edileuza de Fátima Rosina Nardi, Maria Eduarda Pascoaloto da Silva, Leticia de Castilho Peralta, Maria de Fátima Mantovani, Sonia Silva Marcon

**Affiliations:** I Universidade Estadual de Maringá. Maringá, Paraná, Brasil; II Universidade Federal de Mato Grosso do Sul. Campo Grande, Mato Grosso do Sul, Brasil; III Universidade Federal do Paraná. Curitiba, Paraná, Brasil

**Keywords:** Cuidador, Cuidado Domiciliar, Idoso, Saúde da Família, Teoria de Enfermagem.

## Abstract

**Objetivos:**

identificar os estressores experienciados por cuidadores informais à luz do Modelo de Sistemas de Betty Neuman.

**Métodos:**

pesquisa descritiva, de abordagem qualitativa, realizada com cuidadores informais de idosos cadastrados e assistidos por uma instituição em Presidente Prudente, São Paulo. Os dados foram coletados em outubro de 2023, por meio de entrevistas semiestruturadas audiogravadas, e submetidos à análise de conteúdo temática.

**Resultados:**

as 12 cuidadoras participantes tinham entre 30 e 59 anos. Condições como privação de sono, exaustão física, ausência de apoio, dificuldades econômicas e comprometimento da própria saúde constituem exemplos de estressores vivenciados, os quais impactavam a capacidade da cuidadora de enfrentar as demandas do cuidado sem comprometer sua saúde física e emocional.

**Considerações Finais:**

intervenções que promovam o equilíbrio, a saúde mental e o fortalecimento da rede de apoio das cuidadoras podem auxiliá-las a encontrar e utilizar forma mais efetiva, mecanismos de enfrentamento e autorregulação.

## INTRODUÇÃO

No Brasil, entre os anos de 2010 e 2022, a população idosa aumentou em 57,4%, alcançando um total de 32,1 milhões de indivíduos com 60 anos ou mais, o que representa 15,8% da população total^(1)^. Globalmente, a população com 65 anos ou mais ultrapassou 10% em 2023, e pode chegar a 16% até 2050^(2)^. Esses dados constituem desafios econômicos e sociais, uma vez que o aumento da expectativa de vida, sobretudo quando acompanhado de hábitos e comportamentos de risco em relação à condição de saúde, pode levar à senilidade e, consequentemente, à dependência de cuidados diários^(3)^. Ademais, a ocorrência de causas externas, especialmente na população adulta, também tem sido responsável por deficiências físicas ou cognitivas e pela consequente dependência de cuidados, o que aumenta a demanda de suporte constante e atenção especializada^(4)^.

Por vezes, as ações de cuidado são ofertadas no ambiente familiar. Entretanto, restrições financeiras, além do compromisso moral e afetivo, frequentemente levam as famílias a optar por cuidadores informais, geralmente familiares sem preparo psicológico ou treinamento formal^(5)^. Além disso, questões culturais, falta de acesso a recursos de apoio e ausência de políticas públicas efetivas para suporte ao cuidador ampliam os desafios enfrentados por essas pessoas no cotidiano^(6)^.

A função de cuidar de um familiar exige ajustes na rotina e enfrentamento de dificuldades que, sem suporte adequado, podem prejudicar a saúde física e mental do responsável pelos cuidados. Esses prejuízos se manifestam na forma de estresse, sobrecarga e negligência do autocuidado^(7)^, contribuindo para o surgimento ou agravamento de doenças. Ademais, as condições de saúde do cuidador podem comprometer a qualidade dos cuidados prestados^(7)^.

As políticas públicas brasileiras destinadas aos cuidadores informais ainda apresentam caráter limitado e fragmentado. Enquanto o Benefício de Prestação Continuada oferece suporte indireto, iniciativas isoladas, como o programa “Cuidar de Quem Cuida”, em Florianópolis, destacam-se por fornecer apoio psicológico e capacitação técnica^(8)^. O Sistema Único de Saúde (SUS) também contribui por meio de equipes de atenção básica e Centros de Atenção Psicossocial (CAPS), embora a ausência de padronização nacional para ações de capacitação evidencie lacunas importantes. Nesse cenário, a proposta de criação da Política Nacional de Cuidados, ainda em tramitação, reforça a necessidade de avanços mais consistentes nessa área^(9)^.

Nesse contexto, o Modelo de Sistemas de Betty Neuman emerge como um modelo holístico que compreende o ser humano como um sistema aberto, que interage constantemente com o ambiente, e é protegido por linhas de defesa que favorecem a resistência contra estressores internos e externos. Fundamentada nos níveis de prevenção primária, secundária e terciária, o modelo propõe não apenas a promoção da saúde, mas também a minimização dos impactos dos estressores e a restauração do equilíbrio^(10)^, por meio da implementação de intervenções estruturadas e integradas com redes de apoio, políticas públicas e práticas de autocuidado. Quando utilizada de forma adequada, permite a identificação e o atendimento, de maneira abrangente, das necessidades dos indivíduos, inclusive enquanto cuidadores informais.

No Brasil, os cuidadores informais enfrentam desafios significativos, amplificados por fatores como sobrecarga emocional associada à falta de preparo e de suporte adequado, conflitos familiares, e ausência de reconhecimento social, que se constituem como estressores interpessoais. Por sua vez, a inexistência de políticas públicas abrangentes e a insuficiência de recursos financeiros representam estressores extrapessoais, os quais potencializam os impactos negativos na saúde desses indivíduos^(11)^.

Nesse cenário, a abordagem sistêmica oferece uma perspectiva relevante ao considerar que as barreiras de proteção podem ser comprometidas pela falta de estratégias de apoio ou ações preventivas, ocasionando desequilíbrios que resultam em condições adversas de saúde física e mental^(10)^. Conhecer os fatores estressores enfrentados por esses cuidadores permite compreender como eles impactam suas defesas no âmbito físico, emocional e social^(12)^. Além disso, esse conhecimento pode subsidiar a formulação e implementação de estratégias de intervenção que envolvam apoio social, emocional e educacional, favorecendo a restauração e equilíbrio do cuidador, de modo a prevenir o desenvolvimento de doenças e promover sua saúde e bem-estar^(13)^.

## OBJETIVOS

Identificar os estressores experienciados por cuidadores informais à luz do Modelo de Sistemas de Betty Neuman.

## MÉTODOS

### Aspecto éticos

O estudo foi realizado em conformidade com os princípios éticos da Resolução nº 466/2012, e foi aprovado pelo Comitê de Ética em Pesquisa com Seres Humanos da instituição signatária. Todos os participantes manifestaram anuência em participar do estudo mediante assinatura do Termo de Consentimento Livre e Esclarecido. Para assegurar-lhes o anonimato, na apresentação dos resultados, os extratos de suas falas estão identificados com a letra “E”, designando entrevistado, seguida de um número indicativo da ordem de inclusão no estudo.

### Referencial teórico-metodológico

O Modelo de Sistemas de Betty Neuman integra conceitos de sistemas abertos, psicologia e fisiologia, e compreende o ser humano como um sistema holístico, em constante interação com o ambiente e vulnerável a estressores que afetam seu equilíbrio^(10)^. No Brasil, sua aplicação nas pesquisas em enfermagem ganhou destaque na década de 1990, sobretudo no campo da saúde coletiva, tornando-se uma ferramenta relevante para análise de fatores de estresse e planejamento de intervenções de cuidado^(14)^.

O modelo classifica os estressores em intrapessoais, interpessoais e extrapessoais, facilitando a identificação de necessidades específicas e a implementação de intervenções que fortaleçam a capacidade de enfrentamento do indivíduo^(10)^. Na prática profissional, sua aplicação ocorre por meio de uma avaliação sistemática das condições do paciente e da elaboração de planos personalizados voltados à redução dos estressores e à promoção do bem-estar^(14)^.

### Tipo de estudo

Estudo descritivo, de abordagem qualitativa, que precedeu a implementação de uma intervenção de enfermagem baseada no Modelo de Sistemas de Betty Neuman voltada a cuidadores informais de idosos dependentes. Para a redação do relatório do estudo, foram utilizados critérios estabelecidos no *COnsolidated criteria for REporting Qualitative research* ^(15)^ como ferramenta de apoio.

### Cenário do estudo

O estudo foi realizado nos domicílios de idosos dependentes de cuidados, assistidos por uma instituição pública localizada em um município do estado de São Paulo. Essa instituição oferece serviços de saúde ambulatorial e domiciliar, além de atividades voltadas à promoção da socialização, autonomia, autocuidado e autoestima por meio da academia de promoção da saúde. O Serviço de Atendimento Universal Domiciliar ao Idoso, conhecido como S.A.U.D.I., é destinado a pessoas acamadas ou com mobilidade reduzida.

Mensalmente, são realizados cerca de 800 atendimentos de saúde. Para isso, a instituição conta com uma equipe multiprofissional composta por dois assistentes sociais, quatro enfermeiros, cinco fisioterapeutas, três nutricionistas, um fonoaudiólogo, dois psicólogos, três médicos geriatras, um farmacêutico, dois profissionais de educação física e três auxiliares de enfermagem.

### Procedimentos metodológicos

Os participantes do estudo foram 12 cuidadores informais de idosos dependentes, selecionados por conveniência durante visitas domiciliares realizadas pela equipe de saúde no período de agosto a setembro de 2023. O convite para participação no estudo só foi realizado após a pesquisadora principal ter participado de, no mínimo, duas visitas, ou seja, quando já existia uma relação mínima estabelecida entre ela e a cuidadora.

Os critérios de inclusão previamente definidos foram ter idade mínima de 18 anos, atuar como cuidador informal do idoso dependente há pelo menos três meses e apresentar capacidade autodeclarada para compreender e responder ao instrumento de coleta de dados. Não foram instituídos critérios de exclusão.

### Coleta e organização dos dados

Os dados foram coletados em outubro de 2023, mediante entrevistas audiogravadas, após consentimento, previamente agendadas, conforme preferência dos participantes e disponibilidade da pesquisadora. As entrevistas foram realizadas sem a presença de membros da equipe multiprofissional de saúde ou do paciente assistido. Tiveram duração de 60 a 120 minutos, sendo que cada participante foi entrevistado uma única vez.

Durante as entrevistas, foi utilizado um roteiro composto por duas partes: a primeira abordava características sociodemográficas e de saúde do cuidador; a segunda tratava de aspectos relacionados à função de cuidador, como tempo dedicado à atividade, o grau de dependência do idoso, as dificuldades enfrentadas no cotidiano e as estratégias utilizadas para superá-las.

Todas as entrevistas foram conduzidas pela mesma pesquisadora, enfermeira, mestre em saúde da família e doutoranda em enfermagem, com experiência prévia na condução de pesquisas qualitativas e que mantinha apenas contato profissional com as participantes.

### Análise dos dados

As entrevistas foram transcritas na íntegra, preferencialmente no mesmo dia de sua realização, e após submetidas à análise de conteúdo temática, seguindo as três etapas propostas^(16)^. Na pré-análise, os dados foram organizados e submetidos à leitura flutuante para identificação inicial de aspectos relevantes. Na etapa subsequente, exploração do material, foi realizada uma leitura detalhada, seguida pela codificação dos dados em sete códigos, os quais foram agrupados em três núcleos de sentidos. Por fim, na etapa de tratamento dos resultados obtidos e interpretação, após a classificação e agregação dos códigos, emergiram uma categoria e três subcategorias, que foram interpretadas e discutidas à luz do Modelo de Sistemas de Betty Neuman^(10)^, sendo realizadas inferências em relação aos achados.

As entrevistas transcritas não foram validadas pelas participantes, devido à sobrecarga das atividades domésticas e das demandas de cuidado.

## RESULTADOS

Participaram do estudo 12 cuidadoras com idades entre 30 e 59 anos, sendo oito filhas e quatro noras dos idosos dependentes de cuidados. Cinco eram casadas, quatro, solteiras, duas, divorciadas e uma, viúva. Duas participantes estavam afastadas de suas atividades laborais por questões de saúde, enquanto as demais precisaram abandonar seus empregos para assumir os cuidados de seus familiares dependentes, sem perspectiva de reintegração ao mercado de trabalho, por não contarem com uma rede de apoio ou alternativa para assistência ao familiar. A renda familiar de sete participantes variava entre um e dois salários mínimos; quatro viviam com renda de três a quatro salários; e apenas uma vivia com renda de cinco a seis. Destaca-se que nenhuma das famílias recebia benefício social.

Todas as cuidadoras faziam uso de pelo menos um medicamento de uso contínuo. Seis utilizavam um a dois medicamentos por dia; cinco faziam uso de três a quatro; e uma fazia uso de cinco a seis. Quando questionadas sobre sua própria condição de saúde, sete a classificaram como “regular,” três, como “ruim,” uma, como “boa” e uma, como “péssima”, sendo que sete se consideravam estressadas no momento da entrevista. Os problemas de saúde relatados incluíram ansiedade (dez), hipertensão (cinco), depressão (cinco), problemas na coluna (quatro), diabetes tipo 2 (quatro), insuficiência cardíaca (um) e hipotireoidismo (um).

### O lado oculto do cuidado: o desafio físico e mental de cuidar do outro, de si e de sobreviver aos estressores

Esta categoria temática foi estruturada a partir da análise dos dados, agrupando os principais estressores identificados pelas participantes. A codificação dos dados originou três núcleos de sentido, os quais foram organizados em três subcategorias: O conflito do cuidado com o outro como estressor interpessoal; O cuidar de si como desafio: estressores intrapessoais; e A vulnerabilidade financeira como estressor extrapessoal. Tais subcategorias refletem a complexidade do fenômeno do cuidado informal, e foram interpretadas à luz do Modelo de Sistemas de Betty Neuman.

### O conflito do cuidado com o outro como estressor interpessoal

Esta subcategoria expressa as tensões que emergem da relação com o idoso dependente, com os demais familiares e com as exigências da função de cuidar.

A privação de sono, exaustão física resultante das demandas constantes relacionadas à rotina de cuidados, o agravamento de condições de saúde preexistentes e o surgimento de novos problemas de saúde foram estressores referidos pelas participantes.

[...] *chega o final do dia, estou só o pó, sem ânimo para mais nada, nem sempre consigo dar conta de tudo que tem para fazer sabe. A sobrecarga de cuidar dela é grande, e a gente sente o cansaço* [...] *no fim, nem dormir a gente dorme direito.* (E06)

*Depois que ela ficou doente mesmo, eu nunca MAIS soube o que é dormir oito horas na noite* [...] *o máximo que eu durmo numa noite são umas três horas.* (E02)

*Tem dias que ela dorme bem, mas tem dias que não* [...] *como eu durmo no mesmo quarto que ela, eu acordo bem rápido para ver o que ela precisa. Quando eu durmo, tenho medo dela me chamar e eu não acordar, sabe, aí geralmente eu já me deito para dormir com esse receio.* (E10)

*Acho que a condição de saúde dela atual também afetou muito o psicológico dela* [...] *o que consequentemente acabou afetando o meu* [...] *porque tem horas que eu penso que eu não vou aguentar.* (E01)

*Não sobra tempo para fazer as coisas que eu quero. Não sobra tempo* [...] *é dedicação total, somente com ela, só* [...] *nem para o meu marido ultimamente sobra tempo.* (E02)

*O dia passa rápido demais. Quando vejo, ainda estou com roupa de dormir, e já são três horas da tarde, e eu não fiz nada a não ser cuidar dela desde quando me levantei da cama.* (E03)

*Às vezes penso que ela poderia demonstrar mais gratidão por eu ter mudado toda a rotina da minha vida e da minha família para cuidar dela, mas isso não acontece.* (E06)

[...] *conversei com ele ontem* [...] *pai, eu estou cansada fisicamente, emocionalmente, espiritualmente, tudo.* (E03)

### O cuidar de si como desafio: estressores intrapessoais

Os estressores intrapessoais se referem às reações internas desencadeadas pelo exercício da função de cuidadora, incluindo sentimento de frustração, medo de adoecer e impactos na autoestima.

*Ela tem umas atitudes que me estressam muito, e que eu preciso me segurar* [...] *mas que tem hora que aquilo me dá uma angústia muito grande.* (E01)

A condição de assumir o cuidado do outro em tempo integral desestabilizou o sistema de algumas participantes, o que dificultou o autocuidado. Por vezes, a rotina intensa de cuidar do outro gera no cuidador a dificuldade de reservar um tempo para si e a sensação de não ter feito nada, embora tenha feito muito durante o dia.

*Tenho medo de ter problema mental, sabe? Às vezes, a gente vê pessoas dependentes de remédio para dormir* [...] *fiquei com medo de ficar dependente sempre desses remédios.* (E10)

*Te confesso que às vezes tenho medo de ficar louca, é tanta sobrecarga, é exaustivo cuidar* [...] *e eu me sinto muito sozinha. Na verdade, não tenho ninguém nem para desabafar.* (E12)

*Não vou falar para você que eu não saio* [...] *só que eu fico preocupada com ela o tempo todo, então tipo eu acho que a gente não sai, isso é no geral da família. A família não sai mais tranquila, vamos jantar, almoçar fora, não tem* [...] *não conseguimos curtir tanto aquele momento, porque fica preocupada com a pessoa que tá aqui.* (E02)

*Ter um tempo para mim, tipo ir ao salão, é quase impossível* [...] *não tem como eu sair de casa e deixar ela aqui sozinha, e eu não confio em deixar um estranho cuidando dela, tipo* [...] *ela não fala, tenho medo de maus-tratos, sabe* [...]. (E09)

*O tempo que sobra, eu quero descansar, porque eu estou muito cansada. Eu também tenho meus problemas de saúde* [...] *eu preciso descansar, porque senão não aguento, eu vou acabar ficando doente também.* (E02)

Elevados níveis de estresse e frustração resultantes da rotina de cuidado impactam a autoestima do cuidador, o que acaba comprometendo a percepção de valor próprio e bem-estar psicológico.

*Acho que ser cuidador de quem a gente conhece, de um familiar, assim, estressa mais, porque a gente conhece.* (E12)

*Hoje, me sinto mais estressada. Minha autoestima é muito baixa porque antes eu conseguia fazer uma academia, conseguia me cuidar mais, e agora não consigo mais.* (E02)

*Tem dias que fico frustrada em pensar que tenho que fazer tanta coisa por minha mãe e que ela não vai ser mais como era antes. Não importa o que eu fizer, ela não vai voltar a andar, por exemplo. Isso é frustrante* [...]*, todo um trabalho e não ver resultado.* (E10)

De forma semelhante, as dificuldades e adaptações enfrentadas em relação ao aprendizado de novas habilidades, bem como as situações que promovem o crescimento individual ao longo do processo de cuidado de uma pessoa dependente no ambiente domiciliar, foram destacadas.

*Eu não sabia como ela iria evoluir com essa doença, pra ser bem sincera. Fiquei surpresa, porque achei que essa condição avançou de forma muita rápida* [...] *não sei se é assim mesmo, nunca tinha cuidado de uma pessoa com Alzheimer, tudo que eu sei fui aprendendo com ela no dia a dia mesmo.* (E12)

*Eu não sabia nada sobre a doença. Fui vendo vídeos na internet, redes sociais e fui aprendendo. Aos poucos, fui percebendo que cada pessoa pode evoluir de um jeito, e aprendi a adaptar as necessidades na nossa família.* (E09)

*No início, quem mais me ajudou a conhecer sobre essa doença foi as redes sociais. Eu fiquei pesquisando na internet sobre como a doença evoluía, porque não tive esclarecimentos diretos dos profissionais no começo.* (E08)

### A vulnerabilidade financeira como estressor extrapessoal

As participantes ressaltaram que as dificuldades econômicas e os problemas relacionados à rede de apoio constituem estressores extrapessoais que potencializam os desafios enfrentados no cotidiano.

*O nosso gasto aqui é muito grande, né? Não é fácil. A gente se preocupa muito com as contas* [...] *porque tem medicação dela que a gente não pega no SUS. É tudo pela farmácia, né? Então, é muito caro.* (E02)

*Atualmente, não tenho nenhuma renda, porque antes eu conseguia sair para fazer umas corridas particulares. Hoje, raramente eu consigo fazer, tipo, vamos colocar que com isso tenho um ganho de 40 reais mensais* [...] *mas vamos colocar nada que isso não é nada.* (E03)

*Ela só ganha um salário mínimo, então não dá, né, para comprar tudo que precisaria, infelizmente. Tem meses que tenho que escolher o que vou deixar de pagar para tentar não faltar o básico pra ela.* (E05)

Contudo, mesmo diante dos desafios, foram destacadas estratégias utilizadas pelas cuidadoras para mitigar os efeitos do estresse na sua rotina diária.

*Quando estou muito triste, coloco música gospel no meu celular para tentar melhorar, sabe* [...] *quando eu escuto os louvores, parece que renova minhas energias.* (E10)

*Mas quando me sinto estressada, eu oro, eu louvo; eu tenho meu celularzinho, coloco lá, coloco louvor* [...] *faço minhas orações.* (E02)

[...] *quando ela faz algo que me estressa, eu tento fugir do cuidado com ela por um tempinho, saio do quarto para respirar* [...] *mas, às vezes, eu acabo descontando em quem está por perto.* (E06)

## DISCUSSÃO

Os estressores identificados nesta pesquisa, interpretados à luz do Modelo de Sistemas de Betty Neuman, compuseram a categoria temática “O lado oculto do cuidado: o desafio físico e mental de cuidar do outro, de si e de sobreviver aos estressores”. Os achados revelam a complexidade dos desafios enfrentados pelas cuidadoras informais, destacando a predominância feminina nesse papel, especialmente filhas e noras, a renúncia à vida profissional diante da ausência de apoio e os impactos físicos e emocionais decorrentes da rotina intensa de cuidados.

Questões como privação de sono, sobrecarga, busca solitária por informações, ausência de lazer e limitações financeiras agravam ainda mais a vulnerabilidade dessas mulheres. Tais fatores evidenciam a urgência de estratégias estruturadas de suporte social e institucional voltadas ao fortalecimento das redes de apoio e à promoção do bem-estar das cuidadoras. É certo que intervenções dessa natureza não modificariam as condições subjacentes e estruturadas que sustentam esse panorama, mas elas podem minimizar os estressores vivenciados cotidianamente por cuidadores e auxiliá-los a identificar possibilidades de ações que favoreçam o bem-estar e a qualidade de vida.

A organização dos achados em uma única categoria temática com três subcategorias reflete a interdependência entre os tipos de estressores identificados, reforçando a abordagem sistêmica proposta por Neuman. A integração dos núcleos de sentido permitiu visualizar a sobrecarga das cuidadoras como um fenômeno multidimensional, envolvendo aspectos interpessoais, intrapessoais e extrapessoais, que se retroalimentam e comprometem as linhas de defesa do sistema cuidador.

Assim, nesse contexto estruturalmente desafiador, caracterizado pela predominância de cuidadores informais do sexo feminino, faixas etárias específicas, vínculos de parentesco e condições socioeconômicas substancialmente limitantes, a implementação de estratégias destinadas a mitigar os efeitos dessa realidade se torna importante^(17)^. Nesse cenário, o Modelo de Sistemas de Betty Neuman tem como ênfase o contexto de cuidado integral, especialmente utilizado na busca da compreensão da interação dinâmica do homem com o ambiente.

Os estressores interpessoais identificados no presente estudo corroboram os resultados de pesquisa realizada na Coreia do Sul, que destacou o estresse, o desgaste físico e a dedicação intensa de tempo aos cuidados como fatores-chave da sobrecarga de cuidadores informais^(18)^. Esses achados reforçam a urgência de estratégias específicas com enfoque na prevenção secundária para identificação precoce desses fatores de risco à saúde física e mental desses indivíduos, que enfrentam desafios como o cansaço gerado por tarefas diárias, como mobilizar o paciente e administrar medicamentos, além da falta de tempo para autocuidado, agravando a exaustão emocional^(11)^.

Considerando os fundamentos do Modelo de Sistemas de Betty Neuman^(10)^, identifica-se que a necessidade constante de equilíbrio entre responsabilidades familiares e profissionais constitui estressor intrapessoal. Por sua vez, o estresse extrapessoal é proveniente da falta de apoio social e financeiro, que enfraquece as defesas do cuidador, comprometendo sua saúde e o desempenho de seu papel. Estressores interpessoais, por sua vez, decorrem da convivência direta com o familiar dependente e da ausência de reconhecimento pelo esforço da cuidadora, gerando sentimentos de frustração e isolamento emocional. Esses fatores reduzem a eficácia das barreiras protetoras, especialmente em contextos de escassez de apoio do sistema público de saúde, o que intensifica os efeitos da sobrecarga emocional e física. A dificuldade de acesso a medicamentos e aos cuidados necessários agrava ainda mais esse processo, aumentando a vulnerabilidade da cuidadora ao adoecimento e à exaustão.

Segundo Neuman, a ruptura da linha de defesa normal diante de estressores recorrentes exige ações em todos os níveis de prevenção^(10)^. No presente estudo, observou-se que as cuidadoras recorrem frequentemente a estratégias de enfrentamento centradas na fé, no silêncio ou no sacrifício pessoal, o que evidencia a ausência de intervenções estruturadas no nível primário. A falta de suporte formal também compromete as ações de prevenção secundária e terciária, tornando o sistema cuidador mais suscetível à exaustão progressiva.

Na presente pesquisa, os estressores psicológicos associados às condições de saúde do paciente e às demandas constantes do cuidado geram sobrecarga emocional, solidão e temor de colapso mental. O estresse crônico enfraquece a resiliência e dificulta a capacidade de lidar com os desafios do cuidado, contribuindo para sentimentos de ansiedade, medo e impotência diante das responsabilidades acumuladas^(19)^.

Por sua vez, a sobrecarga física e a falta de tempo para o autocuidado, associadas aos desafios interpessoais, como a responsabilidade pelo cuidado do outro e a carência de apoio, também podem impactar significativamente a saúde mental dos cuidadores. A interação entre estressores externos e internos cria um ciclo vicioso, em que a ausência de suporte adequado intensifica o estresse, culminando no adoecimento físico e psicológico do cuidador, e, por conseguinte, ocasiona dificuldade de reconhecer a necessidade de ajuda.

A limitação, ou mesmo a ausência, de apoio aos cuidadores informais não é um problema que ocorre só no Brasil. Estudo fenomenológico conduzido na Ilha da Madeira, em Portugal, também revelou que esses cuidadores convivem com a escassez de assistência domiciliar e de apoio financeiro, além da inexistência de acompanhamento psicológico e programas de capacitação. Os autores destacaram que a ausência de uma rede de suporte adequada pode comprometer a saúde física e emocional dos cuidadores, e intensificar os níveis de estresse vivenciado por esses indivíduos^(20)^.

Nesse sentindo, sugere-se ampliar iniciativas já existentes no Brasil, como as equipes de Atenção Básica e os CAPS, com o objetivo de oferecer suporte emocional mediante a atuação de grupos de apoio e da oferta de terapia psicológica. A expansão dos serviços de assistência domiciliar e de cuidados paliativos no âmbito do SUS, aliada à implementação de programas de descanso e ao fortalecimento das redes comunitárias de suporte, pode contribuir com a promoção da saúde integral dos cuidadores. Além disso, é fundamental ampliar o debate sobre a promoção da saúde mental desses indivíduos, considerando a adoção de ações estratégicas que envolvam orientação sobre questões psicológicas e manejo do estresse. Tais iniciativas precisam contemplar tanto os cuidadores quanto seus familiares, promovendo diálogos abertos sobre as dificuldades vivenciadas e alternativas para fortalecer as linhas de defesa, resiliência e bem-estar geral^(19)^.

A importância do fortalecimento do suporte aos cuidadores informais foi evidenciada em uma intervenção, com jogo educativo como ferramenta de apoio, realizada com 19 cuidadores em Portugal. Os resultados demonstraram eficácia no aprimoramento do suporte emocional e educacional, levando os autores a concluir que a utilização dessa estratégia contribuiu para o fortalecimento das defesas dos cuidadores, resultando em um desempenho mais equilibrado e eficiente nas atividades de cuidado^(21)^.

As participantes da presente pesquisa relataram desvalorização de suas próprias necessidades devido à falta de tempo para si e sensação de insignificância, embora sejam diretamente responsáveis pelas inúmeras demandas exaustivas do cuidado. Esse cenário reflete um ciclo de sobrecarga e autoanulação, resultado dos estressores interpessoais e extrapessoais que desestabilizam a saúde física, emocional e social^(19)^. A priorização constante do outro em detrimento de si intensifica sentimentos de vazio e frustração, revelando um desgaste progressivo das barreiras protetoras, especialmente no que se refere à saúde mental e ao equilíbrio entre vida pessoal e familiar.

As exigências do cuidado prejudicam a realização de atividades prazerosas, como dedicar tempo ao cônjuge e desfrutar de momentos de lazer. A substituição do autocuidado e das interações sociais por tarefas exaustivas afeta diretamente a saúde emocional, enfraquecendo os vínculos interpessoais e familiares. Esse desequilíbrio entre o cuidado de si e do outro compromete os mecanismos protetores do cuidador, aumentando sua vulnerabilidade ao estresse e ao desgaste físico e emocional, dificultando a manutenção do bem-estar e o equilíbrio necessário para enfrentar os desafios do cuidado^(19)^.

Diante desse cenário, promover o autocuidado pode reduzir parte do impacto das exigências do cuidado, embora sua prática dependa de fatores como condição financeira e acesso a recursos. Em contextos mais favoráveis, é possível contratar cuidadores formais e buscar suporte emocional. Já em situações de vulnerabilidade, redes familiares e comunitárias podem oferecer apoio gratuito. Períodos de descanso, redistribuição de tarefas e orientações sobre organização do tempo são estratégias viáveis para fortalecer o bem-estar dos cuidadores, independentemente da condição socioeconômica.

A implementação dessas medidas não apenas fortaleceria a capacidade do cuidador de se proteger dos estressores, mas também contribuiria para a redução dos fatores externos que prejudicam seu bem-estar psicológico e físico^(11)^. Esses aspectos são importantes, pois as cuidadoras em estudo destacaram a complexidade e o desgaste emocional envolvidos no cuidado a um familiar que, apesar de realizado com carinho, traz tensões profundas devido à proximidade e ao vínculo afetivo.

Nesse contexto, a comunicação também desempenha um papel fundamental, uma vez que os cuidadores frequentemente precisam agir por instinto ou dedução, devido à falta de orientação adequada. A transferência de informações, sem considerar o nível de compreensão do cuidador, intensifica a sensação de impotência e frustração, agravando o estresse psicológico. Além disso, a percepção de que seus esforços não resultam em melhorias significativas no cuidado do ente querido contribui para o desgaste emocional, prejudicando a motivação e a capacidade de enfrentamento das adversidades^(22)^.

Muitas cuidadoras enfrentam a dificuldade de se ajustar a novas tarefas, especialmente quando não possuem conhecimento sobre a doença em questão. O aprendizado ocorre a partir da experiência cotidiana e informações adquiridas na internet e redes sociais. Embora seja fundamental, esse processo revela a carência de orientação especializada, sobrecarregando o cuidador com informações a serem assimiladas de maneira autônoma e nem sempre fundamentadas cientificamente. Destarte, um apoio profissional mais direcionado é importante para aprimorar as habilidades e o desenvolvimento pessoal necessário para o cuidado do outro, o que, por sua vez, fortalece a capacidade de lidar com o sofrimento^(23)^.

Nessa direção, promover o autocuidado ajuda o cuidador no enfrentamento dos desafios diários, mas sua viabilidade depende de fatores como renda e acesso a recursos. Famílias com melhores condições podem contratar cuidadores formais, além de poder buscar apoio emocional. Já aquelas em situação de vulnerabilidade podem ou não contar com redes familiares e comunitárias. Destarte, estratégias como descanso, divisão de tarefas e organização do tempo são fundamentais para preservar a saúde dos cuidadores.

As participantes relataram o uso de algumas estratégias para lidar com o estresse diário, como ouvir determinado tipo de música ou mesmo cantar, fazer orações e breves momentos de descanso. Essas práticas, especialmente as espirituais, oferecem apoio emocional, proporcionando uma pausa das tensões do cuidado e permitindo uma reconexão com o sentido de vida e espiritualidade^(24)^. A oração e o louvor foram destacados pelas participantes como recursos que auxiliam na redução do estresse, alívio da tensão e promoção da tranquilidade emocional, funcionando como recursos de enfrentamento que fortalecem a resiliência do cuidador, especialmente no cuidado de familiares com doenças crônicas.

Práticas religiosas tendem a ser muito valorizadas por pessoas mais velhas, especialmente em contextos de saúde. Atualmente, é comum que idosos cuidem de pessoas dependentes, embora essa não tenha sido a realidade das participantes deste estudo. Para idosos de grupos de convivência, a qualidade de vida envolve mais do que alimentação e atividade física - inclui boas relações familiares, espiritualidade e religiosidade que, segundo seus relatos, oferecem conforto, força e esperança, ajudando a enfrentar limitações, perdas e estresse^(25)^.

O afastamento temporário da situação estressante também pode ser visto como uma estratégia adaptativa para preservar a saúde mental^(22)^. Embora a fuga não resolva o problema subjacente, ela oferece ao cuidador um tempo necessário para reequilibrar suas emoções e evitar reações impulsivas que possam prejudicar a relação com a pessoa que recebe os cuidados. Essas estratégias de enfrentamento, que buscam restaurar a energia emocional e mental do cuidador, são fundamentais, especialmente diante da sobrecarga imposta pela rotina intensa de cuidados. Entretanto, elas não são suficientes para mitigar os estressores a longo prazo, o que destaca a importância de intervenções mais estruturadas que ofereçam suporte psicológico e social contínuo, além de cuidados médicos adequados^(26)^.

Essas experiências evidenciam a importância da educação continuada para cuidadores, assim como o acesso a informações claras sobre doenças crônicas e estratégias de enfrentamento^(27)^. Infere-se que a implementação de programas de treinamento e suporte, desenvolvidos em conjunto pela rede de saúde - incluindo Atenção Primária à Saúde, serviços hospitalares e Serviço de Atenção Domiciliar - pode melhorar a qualidade de vida dos cuidadores e das pessoas assistidas. Embora o uso de ferramentas como a internet seja válido, é essencial que esse suporte seja complementado por orientação prática e especializada.

O uso de modelos de análise para identificar os estressores enfrentados pelos cuidadores informais oferece uma oportunidade de transformar um contexto desafiador em uma realidade mais equilibrada e acolhedora. Essa abordagem possibilita a criação de intervenções que garantam a continuidade e qualidade do cuidado, ao mesmo tempo em que protege a saúde e o bem-estar das cuidadoras^(19)^. Com um suporte contínuo e adequado, é possível atenuar os efeitos do estresse crônico e melhorar a capacidade de lidar com as exigências físicas, emocionais e sociais dessa função.

Essas medidas, ao reforçar os mecanismos de enfrentamento, contribuem para restaurar ou preservar a linha de defesa normal do sistema cuidador, promovendo equilíbrio físico e emocional. Conforme o modelo de Neuman^(10)^, esse suporte contínuo pode funcionar tanto no nível primário, ao prevenir o agravamento do estresse, quanto no terciário, ao auxiliar na recuperação do sistema após a crise.

Dessa forma, a consolidação dos achados em uma única categoria temática não representa uma limitação, mas sim uma escolha metodológica fundamentada na interconexão entre os estressores e no caráter sistêmico do cuidado informal. Ao abordar simultaneamente aspectos físicos, emocionais, relacionais e sociais, a análise revela a inseparabilidade dos fatores que impactam a saúde das cuidadoras, reforçando a utilidade do modelo de Neuman como estrutura de análise e intervenção.

### Limitações do estudo

Uma possível limitação do estudo reside no fato de a amostra ter sido composta por apenas 12 cuidadoras, todas localizadas a partir do cadastro do idoso em uma única instituição, o que pode ter limitado a variabilidade na identificação dos estressores e a impossibilidade de validação das transcrições pelas participantes devido à sobrecarga de cuidados.

### Contribuições para a área da enfermagem

O estudo amplia a compreensão sobre os fatores de risco para o estresse dos cuidadores informais de pessoas dependentes em ambiente domiciliar, elucidando as dimensões interna e externa dos estressores vivenciados por essa parcela populacional. Ao focar no impacto emocional, físico e social do cuidado, o estudo promove a visibilidade de um grupo vulnerável, muitas vezes invisibilizado no cuidado à pessoa dependente.

A utilização do Modelo de Sistemas de Betty Neuman para analisar as experiências dos cuidadores oferece uma estrutura teórica robusta que pode ser aplicada em práticas clínicas com vistas à identificação dos estressores e suas consequências nos cuidadores. Esse modelo, ao considerar os diferentes níveis de defesa e mecanismos de resistência, permite à enfermagem desenvolver estratégias de intervenção direcionadas e personalizadas para cada fase de vivência do cuidador.

Ao identificar os principais estressores e suas repercussões para os cuidadores, o estudo contribui para o desenvolvimento de programas de promoção da saúde direcionados a essa população, com enfoque na prevenção do estresse e na melhoria da qualidade de vida. As estratégias de autocuidado e a implementação de intervenções de apoio emocional, por exemplo, podem ser integradas ao trabalho da enfermagem comunitária e domiciliar, tendo impacto direto na saúde dos cuidadores.

## CONSIDERAÇÕES FINAIS

O estudo, embasado no Modelo de Sistemas de Betty Neuman, reafirmou a complexidade dos estressores vivenciados por cuidadores informais e os mecanismos de enfrentamento adotados pelas cuidadoras como estratégias de autorregulação, incluindo a oração e a música. Tais práticas proporcionam alívio emocional, embora se mostrem insuficientes para reduzir significativamente os elevados níveis de estresse, particularmente no que tange à exaustão física e emocional.

A sobrecarga de tarefas, o isolamento social e a falta de suporte financeiro e emocional, somados às dificuldades de saúde física e mental, intensificam a rotina dos cuidadores. A falta de reconhecimento e apoio, aliada ao agravamento de condições como ansiedade e depressão, compromete a capacidade de enfrentamento, gerando uma sensação de frustração e sobrecarga. Esses fatores destacam a necessidade de intervenções que promovam o equilíbrio, a saúde mental e o fortalecimento da rede de apoio dos cuidadores.

É necessário o desenvolvimento de políticas públicas e programas de apoio que ofereçam suporte emocional, orientação sobre manejo do estresse e estratégias de autocuidado. A criação de uma rede de apoio mais robusta, que inclua assistência psicológica, social e financeira, é essencial para reduzir o impacto negativo do cuidado informal e preservar a saúde e o bem-estar dos cuidadores, fortalecendo sua capacidade de enfrentar as demandas do cuidado sem comprometer sua saúde física e emocional.

Sugere-se que estudos futuros tenham como foco a implementação e avaliação de intervenções personalizadas para cuidadores informais, considerando contextos socioculturais e características individuais diversas. Pesquisas sobre resiliência emocional, tecnologias de suporte remoto e grupos terapêuticos são promissores. Também é importante avaliar políticas públicas que ofereçam suporte financeiro e emocional, além da eficácia de práticas integrativas na promoção da saúde mental e bem-estar dos cuidadores. Embora tais intervenções não possuam a capacidade de modificar as condições subjacentes e estruturadas que sustentam esse panorama, podem minimizar os estressores vivenciados cotidianamente por cuidadores e auxiliá-los a identificar possibilidades de ações que favoreçam o bem-estar e a qualidade de vida das cuidadoras.

## Data Availability

Os dados de pesquisa só estão disponíveis mediante solicitação.
